# Restaurant kids’ meal beverage offerings before and after implementation of healthy default beverage policy statewide in California compared with citywide in Wilmington, Delaware

**DOI:** 10.1017/S1368980021001245

**Published:** 2022-03

**Authors:** Lorrene D Ritchie, Laura Lessard, Phoebe Harpainter, Marisa M Tsai, Gail Woodward-Lopez, Tara Tracy, Wendi Gosliner, Kathleen McCallops, Isabel Thompson, Allison Karpyn

**Affiliations:** 1 Nutrition Policy Institute, Division of Agriculture and Natural Resources, University of California, 1111 Franklin Street, Oakland, CA 94607, USA; 2 Department of Behavioral Health & Nutrition, University of Delaware, Newark, DE, USA; 3 Center for Research in Education and Social Policy, University of Delaware, Newark, DE, USA; 4 School of Public Health, University of California, Berkeley, Berkeley, CA, USA

**Keywords:** Children, Beverage, Meal, Restaurant, Policy, Fast food

## Abstract

**Objective:**

In 2019, California and Wilmington, Delaware‘ implemented policies requiring healthier default beverages with restaurant kids’ meals. The current study assessed restaurant beverage offerings and manager perceptions.

**Design:**

Pre-post menu observations were conducted in California and Wilmington. Observations of cashiers/servers during orders were conducted pre-post implementation in California and post-implementation in Wilmington. Changes in California were compared using multilevel logistic regression and paired *t* tests. Post-implementation, managers were interviewed.

**Setting:**

Inside and drive-through ordering venues in a sample of quick-service restaurants in low-income California communities and all restaurants in Wilmington subject to the policy, the month before and 7–12 months after policy implementation.

**Participants:**

Restaurant observations (California *n* 110; Wilmington *n* 14); managers (California *n* 75; Wilmington *n* 15).

**Results:**

Pre-implementation, the most common kids’ meal beverages on California menus were unflavoured milk and water (78·8 %, 52·0 %); in Wilmington, juice, milk and sugar-sweetened beverages were most common (81·8 %, 66·7 % and 46·2 %). Post-implementation, menus including only policy-consistent beverages significantly increased in California (9·7 % to 66·1 %, *P* < 0·0001), but remained constant in Wilmington (30·8 %). During orders, cashiers/servers offering only policy-consistent beverages significantly decreased post-implementation in California (5·0 % to 1·0 %, *P* = 0·002). Few managers (California 29·3 %; Wilmington 0 %) reported policy knowledge, although most expressed support. Most managers wanted additional information for customers and staff.

**Conclusions:**

While the proportion of menus offering only policy-consistent kids’ meal default beverages increased in California, offerings did not change in Wilmington. In both jurisdictions, managers lacked policy knowledge, and few cashiers/servers offered only policy-consistent beverages. Additional efforts are needed to strengthen implementation of kids’ meal beverage policies.

Sugar-sweetened beverages (SSB) are a leading contributor to child obesity^(1)^. On any given day, 44 % of 2–5-year-old children in the USA consume SSB^(2)^, and SSB consumption is positively associated with eating in restaurants^(3)^. One-third of children and adolescents aged 2–19 years eat from a quick-service restaurant (QSR) on any given day^(4)^, where 80 % of beverages offered with kids’ meals are SSB^(5)^ and SSB options are increasing^(6)^. Yet, parents and children are receptive to restaurant meals served with milk or water in lieu of less healthy beverages^(7)^. A study in theme park restaurants found that customers accepted kids’ meal healthier default beverages (HDB) two-thirds of the time, instead of requesting an SSB^(8)^.

Requiring restaurant kids’ meals to offer only healthier beverages has been prioritised to improve beverage intakes of children^(9)^. ‘Default’ beverages are automatically included or offered as part of kids’ meals, absent an alternative customer request^(10,11)^. Behavioural economics theory posits that customers will tend to accept defaults rather than make the effort to request a substitution^(12)^. Although several US jurisdictions have adopted HDB ordinances^(12)^, implementation has not been assessed. Small-scale evaluations of voluntary restaurant programmes have shown mixed results^(5,13)^.

Beginning in January 2019, California was the first state and Wilmington the first city in Delaware to adopt an HDB policy. The California law (SB1192) specifies default beverages as only water and unflavoured milk or a non-dairy equivalent (of any fat content)^(10)^; the Wilmington policy (Ord. 18-046) adds flavoured milk and unsweetened 100 % juice or juice diluted with water to California’s options^(11)^. The primary study aim was to assess pre-post change in California and Wilmington restaurants’ implementation of their respective HDB policies. We hypothesised that compared with baseline, after policy adoption restaurants would be more likely to offer policy-consistent beverages with kids’ meals both on menus (in California and Wilmington) and during orders (assessed only in California). A secondary aim was to describe restaurant experiences with policy implementation and to understand differences between statewide policy implementation in California and citywide implementation in Wilmington.

## Methods

SB1192 passed in California in September 2018 and took effect statewide on January 1, 2019. Ordinance 18-046 passed in Wilmington, Delaware, in October 2018 and took effect citywide in mid-January 2019. Longitudinal data on menu boards were collected from a cohort of restaurants the month prior and 9–12 months after HDB policy implementation in both jurisdictions. Pre-post data from orders taken from cashiers were collected in California only. Data collectors received a 2-hour training prior to data collection, and consent was obtained from managers prior to conducting interviews.

### Restaurant selection

In California, all sixty health departments conducting CalFresh Healthy Living work (California’s Supplemental Nutrition Assistance Program-Education, or SNAP-Ed) were invited and eleven county health departments (Butte, Fresno, Madera, Merced, Monterey, Orange, Sacramento, San Joaquin, San Mateo, San Bernardino and Sonoma) chose to participate in the current study. These eleven health departments identified SNAP-Ed eligible census tracts of programmatic interest to their work to improve beverage intake. To qualify for SNAP-Ed, at least half of residents in identified census tracts had incomes < 185 % of the federal poverty level. Dun and Bradstreet data^(14)^ were used to identify all QSR (*n* 205) in these census tracts. Then, based on review of restaurant websites and/or phone calls, we identified those that offered kids’ meals that include a beverage. QSR were defined as restaurants where food is ordered from a relatively limited menu and without table service, though seating may be provided^(15)^. Although all restaurants that offer a kids’ meal with a beverage are subject to SB1192, only QSR were included due to their high prevalence in lower income neighbourhoods^(16)^, their high proportion of total kids’ meal sales^(17)^ and because QSR are a common source of added sugars in the diets of Americans^(18)^.

The 205 California QSR were grouped into geographic clusters within each county. Clusters were ordered to minimise travel time and maximise the number of QSR that could be reasonably visited during the available 3-week period in December 2018 (after passage of the legislation in September 2018 and all preparatory activities completed but before the policy took effect in January 2019). Within each cluster, QSR were stratified according to with/without voluntary standards and then ordered randomly within each stratum. QSR with ‘voluntary standards’ committed to offer various combinations of milk and/or chocolate milk, juice and water as healthier defaults with kids’ meals prior to any policy enactment^(19)^. Clusters and restaurants within clusters were visited in the specified order described above from 11.00 to 19.00 on weekends and from 14.00 to 19.00 on weekdays for a maximum of 2·5 h or when five customer surveys were obtained (customer survey data not reported in this paper due to inadequate sample attained). Within these allotted time frames, data collectors were able to visit 126 restaurants (61 % of the selected sample); of these, fifteen were excluded for not offering a kids’ meal that included a beverage (*n* 4), not being a QSR (*n* 1) or both (*n* 10). Of the 111 remaining, 110 had an observation conducted pre- and post-policy. The observation at one QSR was not completed because a drive-through operator mistakenly reported kids’ meals were not available.

In Wilmington, Delaware, all restaurants within city limits (*n* 176), generated by Standard Industrial Classification codes^(20)^, were initially included in the sample. Restaurants were then categorised as QSR or full-service restaurants. Full-service restaurants were defined as restaurants where customers sit at a table and order from a server or wait staff^(15)^. After visits or phone calls, six QSR and ten full-service restaurants were identified as serving kids’ meals bundled with a beverage; the remaining restaurants either did not serve a kids’ meal (*n* 81), were not a restaurant (*n* 34), served a kids’ meal that was not inclusive of a beverage (*n* 19) or were not reached to confirm eligibility after three attempts (*n* 15). In between the pre- and post-policy observations, one restaurant closed and another restaurant stopped offering a bundled kids’ meal, for a final sample for comparison of fourteen.

### Restaurant observations

A restaurant observation tool was developed, field-tested and revised by a team of social, behavioural, public health researchers and registered dietitians^(19)^. The observation tool was used by trained research staff and university student volunteers to collect data on kids’ meal beverages featured on menus (or menu boards) and how cashiers/servers offered beverages with kids’ meals during the data collector’s order. Data were collected inside the restaurant and in a drive-through window when present. In California, meal orders were placed during both pre- and post-policy visits; in Wilmington, meal orders were placed post-policy only.

Beverages on menus were recorded as

- Unsweetened water (tap water served from a water pitcher or as an option at the soda fountain and/or unsweetened bottled water);
- Unflavoured milk (or non-dairy equivalent);
- Flavoured milk;
- Unsweetened juice (100 % fruit juice and water-diluted juice);
- SSB (soda, sports drink, energy drink, fruit-flavoured drink, fountain drink(s) not further specified, horchata (a traditional drink made from soaking rice in water and adding sugar, spices and/or nuts), milk shake and coffee or tea with added sugars);
- Diet drink (soda, tea and other beverages with low or no calorie sweeteners added);
- ‘Drink’/‘Kids’ drink’, not further specified.

Milk fat content was recorded if shown. After observing the menu, researchers placed a kids’ meal order in available venues (drive-through and/or inside the restaurant) using a standardised script (kids’ meal was requested without specifying the beverage) to document which beverages were verbally offered initially with the meal by the cashier/server. Kids’ meal price or price range was recorded from menu boards inside.

Both the California and Wilmington policies specify the default beverages that can be ‘offered’ and included on the menu but provide scant other guidance on policy implementation beyond stating that other beverages can be requested by customers and sold with kids’ meals. The stated intent of the policy, however, is to support child consumption of healthier beverages. We therefore chose to define optimal policy implementation based on (1) restaurant menus listing only default beverages with kids’ meals and (2) restaurant staff verbally offering only default beverages with kids’ meals at the point-of-sale. Optimal policy implementation for menus (inside the restaurant and/or at the drive-through window) was defined as featuring (written and/or pictured) the following beverages (consistent with the respective laws in each jurisdiction):

- only water and/or unflavoured milk in California;
- only water, any type of milk (unflavoured or flavoured) and/or unsweetened juice in Wilmington.

Optimal policy implementation for kids’ meal orders was defined as cashiers/servers initially offering only policy-consistent beverages (inside the restaurant and/or at the drive-through window). Therefore, restaurant-level policy implementation measures included assessments of menus and ordering both inside the restaurant and at drive through windows (when available), thereby including a total of up to two measures of menus and two measures during ordering for each restaurant.

### Manager interviews

Researchers attempted to interview a manager at each restaurant after completing the post-policy observation. If a manager was not available to complete the interview in person, researchers requested a phone number. Up to five attempts at calling the manager were made. In California, seventy-five managers were reached and participated in the interviews, two were conducted by phone (one fully; one partially in-person and partially by phone); in Wilmington, all fifteen managers were interviewed in person. Responses were manually recorded by researchers on an interview script. Each interview took 10–15 min to complete.

Managers were asked for their title (owner [independent or franchise], manager [but not owner] or other; hereafter all referred to as manager) and when they began working at that restaurant. If employed at the restaurant prior to January 2019, managers estimated current sales of kids’ meals and percent of kids’ meals sold with specific beverages, as well as sales prior to the policy, a method used previously as a proxy when sales data are not obtainable^(21)^. Managers were then asked about the policy itself (knowledge, support and related customer complaints), and perceived implementation facilitators, including provision of information or training, and barriers.

### Data analysis

Power calculations in California indicated that with a sample size of 110 (eight chains with ≥ 2 restaurants and eleven independent restaurants; alpha of 0·05, power of 0·80, intraclass correlation of 0·1 and initial proportion with policy-consistent implementation of 6 %), we were powered to detect an 18 % increase in menu boards that were policy consistent. Power calculations were not performed for the census sample of Wilmington restaurants. Descriptive statistics including proportions, means and sd were calculated. Pre-post policy differences in the proportion of total menus and orders (combining drive-through and inside) offering specific beverages, and overall restaurant-level menus and orders offering only policy-consistent beverages were compared using multilevel logistic regression adjusting for clustering by chain and by restaurant and adjusting for presence of a drive-through. Paired *t* tests were used to compare pre- and post-policy changes in reported sales of meals and beverages adjusting for clustering by chain. Analyses were conducted using Statistical Analysis System software (version 9.4, SAS Institute Inc.). All statistical tests were two-sided, and *P*-values of < 0·05 were considered statistically significant.

## Results

### Restaurant characteristics

Of the 110 QSR observed in California, three-quarters included a drive-through, most (90·9 %) were a chain restaurant (defined as having locations across multiple states) and nearly two-thirds had voluntary kids’ meal beverage standards adopted by their affiliated chain prior to the statewide policy taking effect (Table 1). As described elsewhere, all restaurant voluntary standards allowed more beverage options than the HDB policy in California^(19)^. Of the fourteen restaurants observed in Wilmington, nine were QSR, three of which had drive-through windows; the remaining five were full-service restaurants. Over half of Wilmington restaurants were chains and three had voluntary standards in place prior to Wilmington’s ordinance. The average cost of kids’ meals was $4·67 in California and $5·37 in Wilmington. A majority of the interviews were completed by a restaurant manager (81·3 % of the seventy-five interviews in California, 66·7 % of the fifteen interviews in Wilmington), and a majority (85·3 % in California and all in Wilmington) had worked at the restaurant prior to January 2019 when the HDB policies took effect.

**Table 1:** Characteristics of restaurants sampled in California (*n* 111) and Wilmington, Delaware (*n* 16)

Observation data	11 Counties, California*		Wilmington, Delaware†	
	*n*	%	*n*	%
Restaurant type				
Quick-service‡	110	100·0	9	64·3
Full-service§	0	0·0	5	35·7
Ordering venues available				
Drive-through and inside	80	72·7	3	21·4
Inside only	29	26·4	11	78·6
Drive-through only	1	0·9	0	0 (0)
Other restaurant attributes				
Chain||	100	90·9	8	57·1
Voluntary beverage standards in place prior to policy¶	70	63·6	3	21·4
Price of kids’ meal				
Mean US$	4·67		5·37	
sd	0·76		1·40	
Manager Interview Data**	*n* 75		*n* 15		
Completed interview					
Manager	61	81·3	10	66·7
Owner	6	8·0	3	20·0
Other	8	10·7	2	13·3
Time at current restaurant					
Before policy went into effect	64	85·3	15	100·0
After policy went into effect	11	14·7	0	0·0
Community Demographic Data††	*n* 111		*n* 16		
Urbanicity‡‡^(27,28)^					
Urbanised area	102	91·9	16	100·0
Urbanised cluster	4	3·6	0	0·0
Rural	5	4·5	0	0·0
Households below federal poverty level^(29,30)^				
Mean %	23·6		22·9	
sd	10·3		4·3	
Adults over 18 years old with less than high school education (% of adult population)^(31,32)^				
Mean US$	32·4		28·1	
sd	14·5		11·8	
Population race/ethnicity^(33,34)^				
Hispanic				
Mean US$	63·3		10·2	
sd	22·7		1·3	
Non-Hispanic White				
Mean US$	24·1		29·2	
sd	19·9		0·4	
Non-Hispanic Asian				
Mean US$	6·2		1·2	
sd	7·2		0·4	
Non-Hispanic Black				
Mean US$	3·5		57·4	
sd	4·4		1·6	
Non-Hispanic other race				
Mean US$	3·0		0·2	
sd	2·8		0·2	
Children^(35,36)^					
< 6 years old				
Mean US$	8·7		8·5	
sd	2·6		0·7	
6–11 years old				
Mean US$	9·4		7·5	
sd	2·9		0·5	

* In California, 110 observations and seventy-five manager interviews were completed across 111 restaurants in eleven counties (Butte, Fresno, Madera, Merced, Monterey, Orange, Sacramento, San Joaquin, San Mateo, San Bernardino and Sonoma); in one restaurant, a manager interview was completed but the observation was not because a cashier mistakenly said that a kids’ meal was not available.

† In Wilmington, Delaware, fourteen observations and fifteen manager interviews were completed across the sixteen restaurants.

‡ Quick-service defined as any restaurant where food is ordered from a drive-through window or inside counter from a relatively limited menu and without wait staff, though seating may be provided.

§ Full-service defined as any restaurant where customers sit at a table and order with a server.

|| Chain defined as any restaurant with locations across multiple states. Independent restaurants (e.g. small regional chains and/or single location) were categorised through evaluation of the restaurant website or restaurant menus posted online.

¶ All voluntary beverage standards included as the default options with kids’ meals: combinations of milk (flavoured and unflavoured low-fat, fat-free or reduced-fat), 100 % juice or diluted juice and/or bottled water.

** One manager in California declined to answer.

†† ‘Community’ is the census tract where the restaurant was located.

‡‡ Urbanised area, ≥ 50 000 people; urbanised cluster, ≥ 2500 but < 50 000 people; rural, < 2500 people in census tract.

In the California sample, most restaurants (92 %) were located in an urban census tract (> 50 000 people). Restaurants in California were located in census tracts with an average of nearly one-quarter of households living below the federal poverty level, one-third of adults with less than a high school education, and 63 % of the population identifying as Hispanic. In the Wilmington sample, all restaurants were located in an urbanised area with nearly one-quarter of households living below the federal poverty level, over one-quarter of the adults with less than a high school education and almost 60 % of the population non-Hispanic Black.

### Beverages on menus

In the California QSR, unflavoured milk and water were the most common beverages listed with kids’ meals on menus (combining inside and drive-through menus) both before (78·8 % of restaurants listed unflavoured milk, 52·0 % of restaurants listed water) and after (78·0 % for milk, 60·0 % for water) the beverage policy took effect (Table 2). The pre-post policy increase in the proportion displaying water was significant (+8 %, *P* < 0·0001). Although no pre-post change was observed for unflavoured milk overall, when examined by fat content, unflavoured 1 %/nonfat milk increased from 17·8 % to 48·0 % of restaurants (*P* < 0·0001), while unflavoured milk without specification of fat content decreased from 65·9 % to 40·7 % (*P* < 0·0001) (data not shown). Significant pre-post declines also were observed for juice (p<.0001), SSB (*P* < 0·0001) and unspecified drink/kids’ drink (*P* = 0·003) on menus in the California sample.

**Table 2:** Beverages included with kids’ meals on menus and offered by cashiers/servers during orders at restaurants before and after policy went into effect in California (*n* 110 quick-service restaurants; *n* 190 inside and drive-through menus and orders) and Wilmington, Delaware (*n* nine quick-service and five full-service restaurants, *n* 17 inside and drive-through menus and orders)*

	Pre-Policy†		Post-Policy†		Pre-Post Change‡		
	*n*	%	*n*	%	*n*	Percentage point	*P*-value
California Menu Boards§							
*Water* || (*n* 125 menus)	65	52·0	75	60·0	+10	+8·0	< 0·0001****
*Unflavoured milk* ¶ (*n* 132 menus)	104	78·8	103	78·0	−1	−0·8	0·65
Flavoured milk¶ (*n* 114 menus)	25	21·9	3	2·6	−22	−19·3	< 0·0001****
Juice** (*n* 125 menus)	56	44·8	13	10·4	−43	−34·4	< 0·0001****
SSB†† (*n* 117 menus)	41	35·0	7	6·0	−34	−29·1	< 0·0001****
Diet drink (*n* 116 menus)	5	4·3	0	0·0	−5	−4·3	n/a
“Drink’/’Kids’ drink’‡‡ (*n* 134 menus)	18	13·4	11	8·2	−7	−5·2	0·003***
California Cashier Orders							
Beverages initially offered by cashier compared with California standards (*n* 180 orders)							
Only *water* and/or *unflavoured milk*	6	3·3	1	0·6	−5	−2·8	< 0·0001****
Other drinks in addition to *above*	18	10·0	34	18·9	+16	+8·9	0·002***
Only other drinks	22	12·2	21	11·7	−1	−0·6	0·77
Asked what drink the customer wanted with kids’ meal	101	56·1	96	53·3	−5	−2·8	0·56
Cashier provided a beverage or fountain cup without offering any options (cashier-chosen beverage)	33	18·3	25	13·0	−8	−4·4	0·15
Wilmington Menus or Menu Boards							
*Water* || (*n* 10 menus)	4	40·0	3	30·0	−1	−10·0	n/a
*Unflavoured milk* ¶ (*n* 12 menus)	8	66·7	7	58·3	−1	−8·3	n/a
*Flavoured milk* ¶ (*n* 11 menus)	2	18·2	2	18·2	0	0·0	n/a
*Milk combined, unflavoured and flavoured* §§ (*n* 12 menus)	8	66·7	8	66·7	0	0·0	n/a
*Juice* ** (*n* 11 menus)	9	81·8	6	54·6	−3	−27·3	n/a
SSB†† (*n* 13 menus)	6	46·2	5	38·5	−1	−7·7	n/a
Diet drink (*n* 13 menus)	6	46·2	3	23·1	−3	−23·1	n/a
‘Drink’/’With Drink’/’Kids’ drink’‡‡ (*n* 10 menus)	3	30·0	3	30·0	0	0·0	n/a
Wilmington Cashier/Server Orders||||							
Beverages initially offered by cashier/server compared with Wilmington, Delaware, standards (*n* 17 orders)							
Only *plain water*, *flavoured or unflavoured milk or juice*	–	–	1	5·9	–	–	–
Other drinks in addition to *above*	–	–	1	5·9	–	–	–
Only other drinks	–	–	1	5·9	–	–	–
Asked what drink the customer wanted with kids’ meal	–	–	8	47·1	–	–	–
Cashier provided a beverage or fountain cup without offering any options (cashier-chosen beverage)	–	–	2	11·8	–	–	–
Not offered drink, had to be requested¶¶	–	–	4	23·5	–	–	–

SSB, sugar-sweetened beverages.

* Beverages in italics are allowed as default beverages per policy. Inside and drive-through venues each represent one observation; results presented combine both venues.

† Pre-policy data were collected within 1 month of the policy going into effect (December 2019–January 2020); post-policy data were collected 9–12 months after the policy went into effect.

‡ Multilevel logistic regression was used to compare pre-post changes, adjusting for clustering by chain and by restaurant and adjusted for presence of drive-through; n/a signifies unable to derive *P* value. ***P < 0·001, ****P < 0·0001.

§ In California, sample size varies due to missing responses. Missing responses were excluded from analysis for that category. In California, data from menus were collected from both inside the restaurant and in the drive-through, where available. In Wilmington, data from menus were collected inside the restaurant only.

|| Water includes tap and/or bottled water that is unsweetened but may include non-energetic flavourings; tap water served from a water pitcher or as an option at the soda fountain was included.

¶ Unflavoured milk includes unsweetened and plain milks or non-dairy equivalents; flavoured milk includes chocolate, vanilla, strawberry or other flavoured milks.

** Juice includes 100 % fruit juice and diluted juice. Diluted juice has water added to 100 % juice without added sugars.

†† Sweetened drinks include soda, sports drinks, energy drinks, fruit-flavoured drinks, fountain drink(s) not further specified, horchata, milk shakes and coffee or teas with added sugars.

‡‡ ‘Drink’ or ‘kids’ drink’ listed on menu without further specification.

§§ Category is displayed for Wilmington only as Wilmington’s ordinance allows any milk (flavoured and unflavoured).

|||| In Wilmington, order data were collected post-policy only.

¶¶ 'Not offered drink, had to be requested’ was a separate response option added for the Wilmington full-service restaurants. The cashier/server did not verbally or physically offer a beverage; the data collector had to request one.

In Wilmington, Delaware, juice was the most commonly featured beverage on 81·8 % of menus pre-policy; decreasing to 54·6 % post-policy (juice is among the default beverages allowed by Wilmington policy). The next most commonly displayed beverages were milk (unflavoured and flavoured combined) (66·7 % pre-policy, 66·7 % post-policy) and SSB (46·2 % pre-policy, 38·5 % post-policy).

### Beverages offered with orders

In California, when an order was placed for a kids’ meal, the most common initial response by cashiers (combining inside and drive-through orders) both pre- and post-policy (56·1 % and 53·3 %, respectively) was to ask what beverage the customer wanted (Table 2). A relatively small, significant decrease (*P* < 0·0001) from six orders (3·3 %) pre-policy to one order (0·6 %) post-policy was observed in cashiers who initially offered only beverages (water and/or unflavoured milk) consistent with the policy. A significant increase in the percent of orders during which cashiers initially offered water and/or unflavoured milk in addition to other non-policy-consistent beverages was observed from 10·0 % pre-policy to 18·9 % post-policy (*P* = 0·002). The percentage of orders during which cashiers initially offered only specific beverages other than water and/or unflavoured milk did not change significantly (12·2 % pre-policy, 11·7 % post-policy).

In Wilmington, researchers did not place kids’ meal orders pre-policy due to funding constraints. Post-policy, the most common initial response by cashiers/servers was to ask what beverage the customer wanted (47·1 %), followed by not offering any drink, in which case the data collector requested a drink (23·5 %). One cashier offered only beverages that were consistent with the policy (5·9 %).

### Optimal policy implementation

**Fig. 1:**
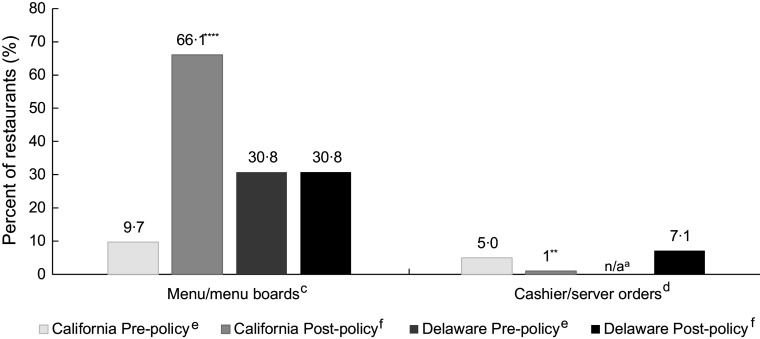
Comparison of restaurant-level policy consistency of menus and orders before and after kids’ meal beverage policy went into effect in California (*n* 78–110 quick-service restaurants) and Wilmington, Delaware (*n* 8–9 quick-service and five full-service restaurants).^a,b^ ^a^Missing responses at pre- or post-policy for inside the restaurant and/or in the drive-through window were excluded from analytic sample resulting in variations in sample size in California. n/a signifies that in Wilmington, data from orders were not collected pre-policy and are reported for inside the restaurant only post-policy. ^b^Multilevel logistic regression was used to compare pre-post changes in California and for Wilmington, Delaware, adjusting for clustering by chain by and adjusting for restaurant type (quick-service or full-service) and presence of drive-through. **P < 0·01, ****P < 0·0001. ^c^Menus consistent with policy if only plain water and/or unflavoured milk with kids’ meal(s) shown on menu boards in California, and if only plain water, any milk and/or any unsweetened juice shown on menus/menu boards in Wilmington, Delaware. ^d^Orders consistent with policy if cashier/server(s) initially offer only plain water and/or unflavoured milk in California and only plain water, any milk and/or any unsweetened juice in Wilmington, Delaware. ^e^Pre-policy data were collected within 1 month prior to the policy going into effect (December 2018–January 2019). ^f^Post-policy data were collected 9–12 months after the policy went into effect (August 2019–December 2019).

Pre-policy, there were few QSR in California where all menus (9·7 %) or all observed cashier orders (5·0 %) offered only beverages consistent with California HDB policy (Fig. 1). Post-policy, there were significantly more restaurants where all menus were consistent with policy (66·1 %, *P* < 0·0001), but significantly fewer where all cashier responses to orders (1·0 %, *P* = 0·002) were consistent with optimal policy implementation. In Wilmington, 30·8 % of restaurants pre- and post-policy had menus that were consistent with the Wilmington policy. Post-policy, 7·1 % of restaurant orders were consistent with optimal policy implementation (orders were not observed at baseline). No restaurants in California or Wilmington were fully consistent with optimal policy implementation for all menus and orders at any time during the study.

### Restaurant manager perceptions

**Fig. 2:**
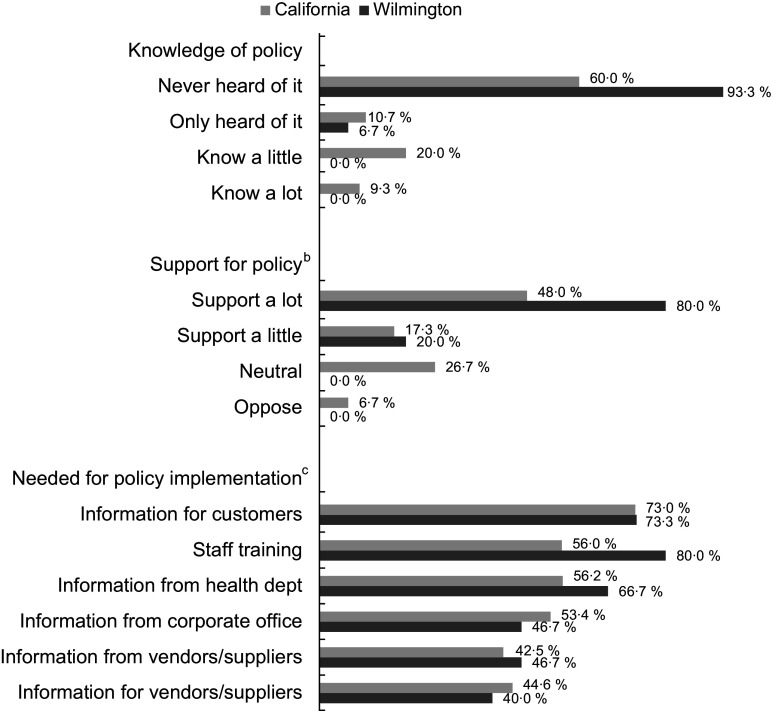
Restaurant manager perceptions of policy in California (*n* 75) and Wilmington and Delaware (*n* 15) after policy went into effect^a^ ^a^Restaurant manager interviews were conducted 9–12 months after policy went into effect (August 2019–December 2019). ^b^Support for policy was asked after the policy was briefly described; one manager in California declined to answer the question. Answer options ‘somewhat oppose’ and ‘strongly oppose’ were combined into ‘oppose’. ^c^Other answer options included: no, not sure or already done. Few managers (3–13% in California, 0–20% in Wilmington, Delaware) reported not sure or already done.

Post-policy, the majority of the managers interviewed in California (60·0 %) and Wilmington (93·3 %) had never heard of the policy (Fig. 2). In California, 20·0 % reported knowing a little about it and 9·3 % reported knowing a lot; none of the managers in Wilmington knew about the policy and only one had heard of it. When the managers in California who knew about the policy (*n* 22) were asked what made policy implementation difficult, the response cited most often was customer preferences (40·7 %), followed by staff training (14·8 %), product availability (14·8 %) and corporate standards or rules (11·1 %) (data not shown). Few managers said vendor contracts (*n* 2) or beverage costs (*n* 1) were a challenge. After the policy was briefly described, a majority of managers strongly or somewhat supported the policy (65·3 % in California, 100 % in Wilmington). Few managers (three in California, one manager in Wilmington) reported any customer complaints about the policy (data not shown). When asked what would help with policy implementation, all options presented were supported by at least 40 % of managers. A majority (73 % each in California and Wilmington) indicated information/promotion for customers would be helpful. Over half of managers in both California and Wilmington supported staff training and information from health departments.

No significant pre- to post-policy changes were reported by managers in the weekly number of kids’ meals sold at the California or Wilmington restaurants (Table 3). Similarly, no significant pre-post changes were reported in the percent of kids’ meals sold with water, milk, juice or SSB.

**Table 3:** Comparison of kids’ meal sales before and after policy as reported by managers at California (*n* 75) and Wilmington, Delaware (*n* 15) restaurants after policy went into effect*

	Pre-policy		Post-policy		Pre-post change		*P*-value†
	Mean	sd	Mean	sd	Mean	sd	
Kids’ meals sold, number/week							
California (*n* 52)	186·0	329·3	201·7	346·0	+15·8	68·6	0·10
Wilmington (*n* 15)	518·0	1085·3	555·9	1137·2	+37·8	94·0	0·12
Kids’ meal beverages sold, % of total							
Water							
California (*n* 58)	3·8	14·9	4·4	15·2	+0·6	4·4	0·29
Wilmington (*n* 15)	11·0	11·4	12·4	13·5	+1·5	3·9	0·18
Milk, mostly unflavoured‡							
California (*n* 58)	7·2	13·5	8·4	15·3	+1·2	11·6	0·20
Wilmington (*n* 15)	5·1	7·8	4·7	7·7	−0·4	1·5	0·35
Milk, mostly flavoured							
California (*n* 58)	14·6	21·0	14·5	20·2	−0·7	10·7	0·97
Wilmington (*n* 15)	20·8	24·2	21·1	23·9	+0·3	1·3	0·35
Juice§							
California (*n* 58)	32·5	31·6	32·9	31·2	+0·4	11·2	0·66
Wilmington (*n* 15)	29·1	25·2	29·1	25·2	0	22·7	1·00
Sugar-sweetened beverage (SSB)||							
California (*n* 58)	41·9	34·6	39·7	33·9	−2·2	18·4	0·38
Wilmington (*n* 15)	34·1	30·8	32·7	27·7	−1·4	23·2	0·82

* Restaurant manager interviews were conducted 9–12 months after policy went into effect (August 2019–December 2019). Sample size varies for each item due to missing responses. All sample sizes refer to number of restaurants where a manager was interviewed.

† Paired *t* tests were used to compare pre- and post-policy changes in reported sales for California and for Wilmington, Delaware, adjusting for clustering for chain. *P* < 0·05 was considered statistically significant.

‡ Respondents who reported any sales of milk were asked if sales were mostly unflavoured, mostly flavoured milk or milk that was not specified to be either flavoured or unflavoured. Responses of mostly unflavoured and milk unspecified were combined.

§ Juice includes 100 % fruit juice and diluted juice (water added to 100 % juice without added sugars).

|| Sugar-sweetened beverage (SSB) include soda, sports drink, energy drink, fruit-flavoured drink, fountain drink(s) not further specified, horchata, milk shake and coffee or tea with added sugars.

## Discussion

To our knowledge, this is the first evaluation comparing restaurants before and after the enactment of kids’ meal healthier default beverage policies intended to promote healthier beverage intake among children. Similar to studies of the effect of voluntary beverage standards adopted by restaurants, we found improvements in some but not all areas^(22)^. In the QSR sampled in California, we observed nearly a sixfold improvement in the proportion of restaurants that displayed only policy-consistent beverages (water or unflavoured milk) on menus post-policy compared with baseline. However, nearly 1 year after policy implementation, one-third of menus at sampled California restaurants were not consistent with the policy. Although the sample size in Wilmington was relatively small, it represented all of Wilmington’s full-service and QSR subject to the policy. There, nearly three-quarters of menus were not consistent with policy. Non-policy-consistent menus were more common in Wilmington than in California, even though Wilmington’s policy includes more beverage options (flavoured milk and 100 % and diluted juice in addition to water and unflavoured milk) than California’s policy. Furthermore, a higher proportion of the sampled restaurants in California (*n* 80, 72·7 %) compared with Wilmington (*n* 3, 21·4 %) had both drive-through and inside menus, which translated into having to meet a higher ‘bar’ of more observed menus to be deemed policy consistent in the California sample. That relatively fewer menus post-policy were consistent with policy in Wilmington compared with California may be explained by the fact that no restaurant managers in Wilmington reported knowing about the policy, compared with nearly one-third of managers knowing a lot or a little about the policy in California. Furthermore, a smaller proportion of Wilmington restaurants was chains. Chains may have corporate-level mechanisms for communicating and implementing policy changes, particularly when those policies are passed statewide in a large state like California. The higher proportion of restaurants in California with voluntary standards may have also contributed to their interest and ability to implement the legislated policy. Previously, we reported that at baseline, a significantly higher proportion of the California sample of QSR with voluntary standards had menu boards that featured only milk, water or unsweetened juice with kids’ meals compared with restaurants without voluntary standards (65·1 % *v*. 4·4 %; *P* < 0·001)^(19)^. Still, due to the stricter policy, many of these California restaurants with voluntary policies were required to change their menus.

While the California and Wilmington policies stipulate that other beverages can be sold with kids’ meals upon customer request, it is important to evaluate whether ordering environments are consistent with stated policy intent to improve healthier beverage intake by children^(10,11)^. We found the kids’ meal ordering experience was rarely consistent with this intent. Post-policy, at only one restaurant each in California and Wilmington did staff initially offer only policy-consistent beverages when researchers ordered a kids’ meal. Offering non-compliant options initially (before a customer asks for options) or initially just asking what drink a customer wants may increase the likelihood that non-compliant beverages are selected, thereby reducing the policy’s effect on child beverage intake.

To give restaurants time to revise menus and adapt operations, we waited 7 months to a year after the policies went into effect before returning to observe restaurants. Although it is possible more time is needed for restaurants to implement the policy fully, given the limited number of managers who reported familiarity with the policy, particularly in Wilmington, full implementation will likely require additional information sharing and/or marketing campaigns. We did not collect data on media coverage of the HDB policies; however, it is possible that restaurant managers become more aware of statewide than local city policies. Encouragingly, a majority of restaurant managers supported the policy and expressed an interest in having more information for customers, more staff training and more policy guidance from their health department. Enforcement efforts also may be needed. It may take additional time for enforcement agencies to develop and implement plans to educate restaurants and monitor compliance.

We also assessed managers’ self-reported sales of kids’ meals and kids’ meal beverages and complaints about the policies in an effort to identify impacts on customer orders as well as address concerns raised about negative impacts of the policy on sales and restaurant viability^(12)^. Restaurant managers did not report changes in the number of kids’ meals nor types of beverages sold in either California or Wilmington or were they aware of many customer complaints about the policy. Past studies have suggested making kids’ meals healthier does not have a negative financial impact^(13)^ and may actually increase sales as consumer demand for healthier options increases^(23,24)^. It is also possible that if policies were more fully implemented, impact on customer complaints, kids’ meal sales and beverages selected with kids’ meals could change.

While HDB policies in California and Wilmington specify that restaurants must display only allowable beverages on menus, how restaurant staff offer default beverages during customer orders is not specified. This lack of specificity may limit policy impact on customer orders. Based on managers self-report, there were no changes in customer orders of water, milk, juice or SSB due to the policy. To be maximally effective in nudging kids’ meal orders to include only healthier beverages, jurisdictions considering future HDB policies should explicitly address the ordering process. In addition, as more restaurants move towards orders that do not involve restaurant staff, such as online ordering (including from third-party vendors)^(25)^ and in-store ordering kiosks^(26)^, these additional ‘menus’ and ordering processes should be addressed in policy language.

The study has several limitations. We did not directly assess changes in consumer orders or child consumption because of HDB policies. Future studies should examine influences on HDB policies’ impact. For example, citywide policies may be less effective than statewide policies due to ease of seeking alternative restaurants outside the legislative jurisdiction. Given our small sample of full-service restaurants, we were unable to evaluate differences by restaurant type. Kids’ meal beverage defaults may be implemented differently and/or have different impacts on consumers in QSR than full-service restaurants^(8)^. For example, water is often served free-of-charge at full-service restaurants, which may influence what beverages customers order with kids’ meals. An additional study limitation is that only 1–2 kids’ meal orders were placed per restaurant by adult researchers usually without children present. It is possible that having children present changes how cashiers/servers offered beverage options with kids’ meals. We did not collect data on which restaurant chains were franchises *v*. corporate-owned, and we did not interview any corporate managers responsible for menus. We also did not assess how information on the HDB policy was disseminated to restaurants. Future studies should examine differential ability of restaurants to change their menus and ordering protocols and how best to disseminate HDB policy information to restaurants, so that appropriate technical assistance and other supports can be provided to support policy implementation. Restaurant managers were likely unable to recall sales data accurately, particularly from 9 to 12 months earlier, minimising our ability to detect small changes in sales; however, given limited policy implementation – particularly during ordering – it is also possible sales had not shifted. Furthermore, manager perceptions may not accurately reflect actual sales. Additional evaluation of sales data using customer receipts or restaurant sales records is needed. Also warranted is documentation of customer feedback under conditions of full implementation of the policy. Finally, future studies should interview managers at the corporate office level as franchise manager may not have a full understanding of the efforts undertaken by the franchise.

Multiple differences existed between the California and Wilmington restaurants that may have contributed to differences in implementation in addition to the policy differences. The sampled restaurants were located in low-income communities in eleven California counties and in one Delaware city, limiting generalisability to other jurisdictions. The California sample included more chains than in Wilmington, which may have made it easier for companies to standardise changes to menus especially since all of a chain’s restaurants in the state were subject to the same policy. While the Wilmington sample was small, it included nearly all restaurants in the city subject to the policy. Finally, the study lacked a comparison state and city without HDB policy, thereby limiting our ability to attribute changes to the policies alone. Given these limitations, additional comparisons are warranted to understand optimal policy implementation and the impact of these policies on child beverage consumption.

In summary, although several US jurisdictions have adopted kids’ meal healthier default beverage policies, little is known about policy implementation. This pre-post evaluation of 2019 policies in California and Wilmington, Delaware, found substantial increases in healthier default beverages being displayed on menus in California QSR, but little change was observed in ordering practices among cashiers/servers in either location. Further, few restaurant managers knew about the policy. Additional support for policy implementation, including restaurant guidance, is needed to ensure that the intent of the policy – an increase in the purchase and consumption of healthier default beverages by children – is achieved.
